# Teacher Emotional Competence for Inclusive Education: A Systematic Review

**DOI:** 10.3390/bs15030359

**Published:** 2025-03-13

**Authors:** Emanuela Calandri, Sofia Mastrokoukou, Cecilia Marchisio, Alessandro Monchietto, Federica Graziano

**Affiliations:** 1 Department of Psychology, University of Torino, 10124 Torino, Italy; emanuela.calandri@unito.it; 2 Department of Political and Social Sciences, University of Salerno, 84084 Fisciano, Italy; smastrokoukou@unisa.it; 3 Department of Philosophy and Education Sciences, University of Torino, 10124 Torino, Italy; cecilia.marchisio@unito.it (C.M.); alessandro.monchietto@unito.it (A.M.)

**Keywords:** inclusive education, disability, special educational needs (SENs), teachers, primary school, secondary school, emotional competence, emotional intelligence (EI), empathy, emotion regulation

## Abstract

Although many studies have examined which teaching strategies are effective in achieving inclusive education, less attention has been paid to the role of teachers’ emotional competence. This study aimed to systematically review the literature on the relationship between teachers’ emotional competence and inclusive education through the following research questions: (1) What aspects of teachers’ emotional competence have been studied in relation to inclusive education? (2) How does teachers’ emotional competence influence different aspects of inclusive education? Five electronic databases were searched for all peer-reviewed empirical studies published from 2010 to February 2025. Studies were selected if they focused on K-12 teachers’ emotional competence in relation to inclusive education and were based on empirical designs. The CASP (Critical Appraisal Skills Programme) checklist was used to assess the quality of included studies. Eighteen studies were included. They drew on partially overlapping definitions of emotional competence (i.e., emotional intelligence, emotional awareness, empathy, and emotion regulation) and considered multiple indicators of inclusion that focused on student (engagement, motivation, emotional self-regulation, emotional development, and academic outcomes) and contextual variables (classroom management, teacher–student relationships, and classroom climate). Outcomes differed across various disabilities and special educational needs (SENs). The role of emotional competence should be considered both in improving teachers’ skills in professional practice and in providing adequate and comprehensive training for future teachers. These findings highlight the need to integrate emotional competence training into teacher education programs and inform education policy aimed at fostering more inclusive learning environments.

## 1. Introduction

### 1.1. Inclusive Education

Since the Convention on the Rights of Persons with Disabilities (CRPD), which was adopted by consensus by the United Nations General Assembly in 2006 and has been ratified by more and more states over the years, inclusive education has been a topic of growing interest in educational and psychological literature. The CRPD recognizes the right to inclusive education for all persons with disabilities, who are defined as “those who have long-term physical, mental, intellectual or sensory impairments which in interaction with various barriers may hinder their full and effective participation in society on an equal basis with others” (CRPD, 2007, p. 4). This approach to disability is similar to that of the International Classification of Functioning, Disability and Health (ICF), which defines disability as “an umbrella term for impairments, activity limitations and participation restrictions. It denotes the negative aspects of the interaction between an individual (with a health condition) and that individual’s contextual factors (environmental and personal factors)” (WHO, 2001, p. 221). General Comment No. 4 on Article 24 of the CRPD (UNCRPD, 2016) defines inclusive education as a comprehensive process of systemic reform. This includes changing educational content, methods, approaches, structures and strategies to remove barriers and provide all students with an equitable and participatory learning experience that meets their needs and preferences (De Beco et al., 2022). The mere placement of students with disabilities in general classes without substantial changes to the organization, curriculum, and pedagogy does not meet the criteria for inclusion (Stelitano et al., 2020).

Parallel to the emergence of the concept of inclusive education, there have been a large number of studies investigating which teaching strategies are effective in achieving this goal. There are now several studies and systematic reviews of teaching strategies for inclusion; in particular, emphasis has been placed on the usefulness of differentiation/personalization of teaching strategies and curriculum adaptation (Amor et al., 2019; Finkelstein et al., 2021; Van Mieghem et al., 2020). However, the majority of these studies focused primarily on the individualization aspects of teaching strategies and placed less emphasis on the role of context, whereas working on the classroom context is a crucial element in achieving inclusion (Naja & Hameed, 2024).

With the advent of Developmental Systems Theory (Ford & Lerner, 1992), contextual factors have been recognized as essential to individual development and cannot be ignored when discussing educational issues. One of the most challenging contextual aspects in terms of implementing inclusion is classroom management, namely “the actions teachers take to create an environment that supports and facilitates both academic and social-emotional learning” (Evertson & Weinstein, 2006, pp. 4–5). It is well known that well-managed classrooms foster meaningful learning and prevent the development of academic and emotional problems for all students, as well as promoting teacher well-being (Chow et al., 2024). One of the most important aspects of classroom management is the ability of teachers to manage relationships between teachers and students and between students and students (Taylor, 2019). While there is a growing pedagogical literature on strategies for managing classroom dynamics to promote inclusion (Farmer et al., 2019; Polirstok, 2015), less attention has been paid to the role of psychological, particularly emotional, competencies of teachers in classroom management, which can promote inclusive education.

### 1.2. Teacher Emotional Competence

Teaching is one of the most emotionally challenging professions, and the role that teachers’ emotional competence plays in successful educational processes is receiving increasing attention; in the meantime, it is necessary to define and delimit the broad construct of emotional competence (Lozano-Peña et al., 2021). Emotional competence includes awareness of emotions, the ability to use and understand emotion-related vocabulary, to identify and recognize emotions in oneself and others, to regulate one’s own emotional states, to express emotions appropriately, also in relation to cultural rules, and to understand the effect emotions can have on oneself and others (APA Dictionary, 2023; Eisenberg & Spinrad, 2004). Many studies have operationalized the concept of teachers’ emotional competence through the construct of emotional intelligence (EI). According to Mayer and Salovey’s model (Mayer & Salovey, 1997), EI comprises four skills: accurately perceiving and appraising emotions; accessing and eliciting emotions when they facilitate cognition; understanding emotional language and using emotional information; and regulating one’s own emotions and those of others to promote growth and well-being. EI thus makes it possible to know how others feel, what they think, and what their intentions are, to understand their emotions and predict their behavior (APA Dictionary, 2023). In particular, teachers’ EI has been shown to be related to a higher ability to motivate students and promote better academic performance (Sayko, 2020), a higher ability to manage classrooms (Valente et al., 2020), as well as better relationships with colleagues, greater enthusiasm for work, and greater personal well-being (Lozano-Peña et al., 2021; Puertas Molero et al., 2019).

Two other psychological constructs that relate to emotional competence and partially overlap with EI are empathy and emotional self-efficacy. Empathy is defined as understanding a person from their frame of reference rather than one’s own, or vicariously experiencing that person’s feelings, perceptions, and thoughts (APA Dictionary, 2023). Empathy particularly emphasizes the aspect of emotional competence related to taking the other person’s point of view (cognitive component) and understanding their emotions (affective component) (Feshbach, 1997; Hoffman, 2008). While a positive relationship has been found between teachers’ empathy and the emotional support given to students, there is still little evidence of a relationship between teacher empathy and the quality of teacher–student interactions and student academic outcomes (Aldrup et al., 2022; Meyers et al., 2019).

Emotional self-efficacy is defined as the perceived ability to regulate and express positive and negative emotions (Bandura et al., 2003). Emotional self-efficacy primarily emphasizes the aspect of emotional competence related to the regulation and expression of one’s emotions, and therefore relates more to the self, whereas empathy relates more to interactions with others (Aldrup et al., 2022). Previous research has found that teachers’ emotional self-efficacy is related to both empathy and teachers’ self-efficacy, emphasizing the role of emotional skills in successfully coping with the challenges of the teaching profession (Goroshit & Hen, 2014; Valente et al., 2020).

While the studies mentioned above generally refer to the emotional competence of teachers in mainstream education, this has been little studied in relation to inclusive education, although teachers’ emotional competence can be a resource for coping with the challenges of inclusive education (Taxer & Gross, 2018). Indeed, teachers have to cope with particularly challenging situations when teaching children with disabilities, special educational needs, or emotional and behavioral disorders, such as students’ uncontrolled emotions, oppositive behaviors and indiscipline, low motivation, and learning difficulties (Silva & Marin, 2019). As a result, it is likely that special education teachers experience more emotionally intense interactions with students compared to general education teachers (Jones & Youngs, 2012). Various aspects of emotional competence can offer a resource for inclusion. For example, the expression of emotions for students with special educational needs (SENs) or disabilities can form an important source of feedback during school activities (Taxer & Frenzel, 2020). The ability to appropriately regulate one’s emotions can impact the ability to cope with externalizing (e.g., aggression) and/or internalizing (e.g., depression, anxiety) issues of students with emotional/behavioral disorders (Kauffman & Landrum, 2018). Finally, empathic ability can enable a teacher to get to know each student and choose the most appropriate way to interact with and support them, and this emotionally supportive environment can promote students’ socio-emotional development (Hamre & Pianta, 2005; Vandenbroucke et al., 2018).

### 1.3. Study Aims

Understanding the role of teachers’ emotional competence in promoting inclusive learning environments requires up-to-date scientific evidence. This systematic review addresses that gap by examining the following research question: How does teachers’ emotional competence improve the implementation of inclusive education? This overarching question was divided into two sub-questions:

(Q1) What aspects of teachers’ emotional competence have been explored in the literature in relation to inclusive education?
(Q2) How does teachers’ emotional competence influence different aspects of inclusive education?

Our review of the existing research on these topics is intended to support both current and prospective educators. Improving the emotional competencies of teachers may allow them to manage a more diverse classroom, to ensure quality inclusive education. It can be concluded that more structured and comprehensive training programs are needed to develop future educators’ emotional competencies. As far as we know, no systematic review has previously provided a comprehensive summary of this topic.

## 2. Materials and Methods

For this systematic review, we followed the recommendations of the PRISMA (Preferred Reporting Items for Systematic Reviews and Meta-Analyses) framework (Page et al., 2021). We registered our systematic review on the Open Science Framework (https://doi.org/10.17605/OSF.IO/XZKYF, accessed on 14 May 2024).

### 2.1. Eligibility Criteria

To be included in our review, studies had to meet the following criteria: (a) focused on teacher emotional competence in relation to inclusive education; (b) involved samples of K-12 teachers (i.e., primary and secondary education); (c) based on empirical quantitative, qualitative, or mixed-methods research designs; (d) written in English; (e) published in peer-reviewed journals; and (f) published since 2010, thus reflecting advances in inclusion research following the adoption of the United Nations Convention on the Rights of Persons with Disabilities (UNCRPD). Studies were excluded for the following reasons: (a) the absence of a focus on teacher emotional competence in relation to inclusive education; (b) a focus on higher education teachers; (c) an exclusive focus on students’ emotional competence; (d) theoretical and conceptual studies without primary empirical research; (e) the use of languages other than English; (f) papers not peer-reviewed (i.e., textbooks, discussion papers, and gray literature); and (g) studies published before 2010. These criteria were employed to refine the selection process and target the most pertinent literature for the review.

### 2.2. Information Sources and Search Query

A systematic search was conducted through the following databases in September 2024: Education Source, ERIC, PsychINFO, Web of Science, and Scopus. An updated search, using the same strategy, was conducted in February 2025.

The key search terms were the following:

#1 Emotional competence OR EI OR emotional regulation OR emotional awareness OR emotional expression OR empathy OR emotional self-efficacy;
#2 K-12 teacher OR primary teacher OR elementary teacher OR secondary teacher OR middle school teacher OR high school teacher;
#3 Inclusion OR inclusive education OR inclusive classroom OR inclusive school OR special education OR special educational needs OR disability;
#4 1 AND 2 AND 3;
#5 Limit #4 to 2010-current; English; peer-reviewed Journal article.

Due to the different database settings, the search strategies and limits were adapted for each database.

### 2.3. Study Selection, Data Extraction, and Quality Assessment

**Figure 1:**
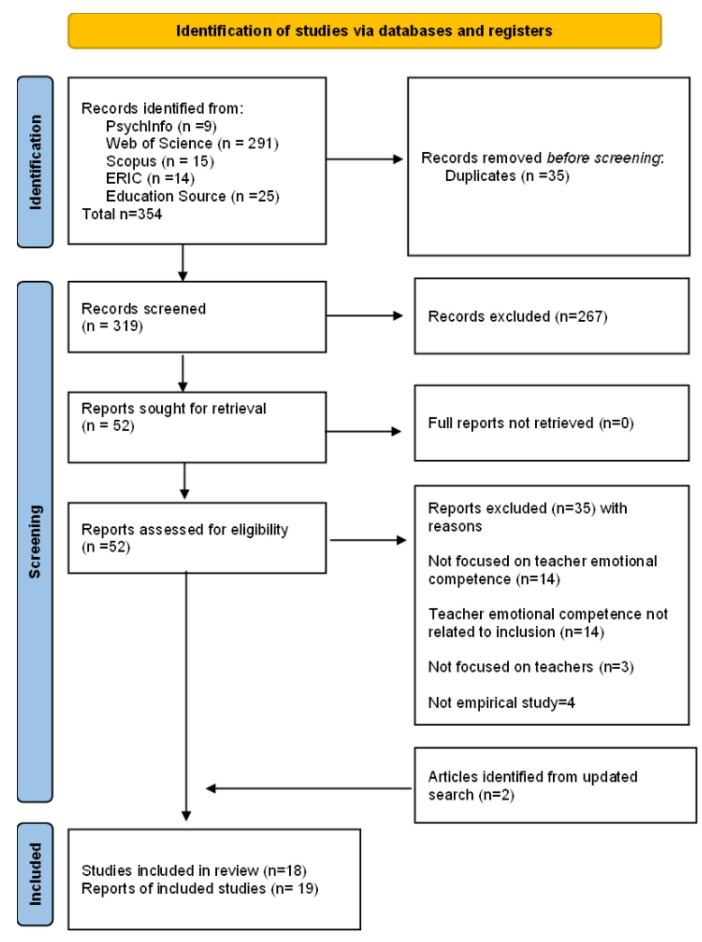
PRISMA flowchart of included studies.

The steps we followed to select relevant studies are illustrated in the PRISMA flowchart in Figure 1. According to PRISMA rules, the flow diagram depicts the different phases of the systematic review and reports the number of records identified, screened, excluded (with reasons for exclusions), and included. The initial database search led to n = 354 records, which were imported into the literature management tool Zotero 7.0 to identify duplicates. After removing 35 duplicates, the remaining 319 records were screened according to the eligibility criteria. Screening of abstracts and titles yielded 52 reports sought for retrieval. After full-text reading, 35 reports were excluded, and 17 reports were included in the review, corresponding to 16 studies (2 reports referred to the same study). Then, 2 additional reports were identified from the updated search in February 2025, leading to a total of 19 reports and 18 included studies. Title, abstract, and full-text screening was performed by two independent reviewers, and in case of disagreement, a consensus was reached by discussion. In case of persistent discrepancies, a third reviewer was involved to make the final decision.

All data relevant to the study aims were extracted from the included studies. Information on the author(s), year, country, study design, teacher sample, grade level, type of student disability/SEN, and overall results were tabulated by one researcher, and another researcher reviewed the extracted data.

The methodological quality of the included studies was assessed using the CASP (Critical Appraisal Skills Programme) checklist for systematic reviews (CASP, 2018). This checklist consists of ten questions divided into three broad sections (Section A: “Are the results of the study valid?”; Section B: “What are the results?”; Section C: “Will the results help locally?”). The checklist acts as a pedagogical tool to determine validity, consistency, methodological quality, presentation of results, and study outcomes. Methodological quality was assessed by two independent reviewers, and disagreements were resolved through discussion to ensure reliability. All selected studies were included in the systematic review regardless of the results of their methodological quality.

## 3. Results

### 3.1. General Characteristics of Included Studies

The included papers were published from 2016 onwards, with the number of publications increasing from 2021 onwards, indicating a current and growing interest in the topic of teachers’ emotional competence for inclusive education. Nine studies were conducted in European countries (two in Belgium, two in Norway, three in Spain, one in Poland, and one in Italy), three in South America (two in Chile and one in Brazil), two in the USA, two in Africa (Nigeria and South Africa, respectively), one in Israel, and one in Australia. In terms of the geographical distribution of the literature, there was a remarkable spread. Most of the selected studies (10) were quantitative in nature, while seven were based on a qualitative methodology (interviews, focus groups, or observations) and one used a mixed-methods design (Table 1).

The sample sizes of the studies ranged from 7 (De Klerk & de Klerk, 2018) to 739 (Graziano et al., 2024), depending on the objective of the study and the methodology used. The grade level in which teachers worked was not reported in all studies; when reported, it ranged from pre-primary to secondary school, and most studies included teachers from primary/elementary school.

Not all studies specified whether they sampled general or special teachers; only one study (Skura & Świderska, 2022) distinguished between teachers in different types of schools in relation to the country-specific school organization (mainstream schools, integrative schools, and special schools); in some studies, teachers were referred to as educators/instructors. Two studies (Arias-Pastor et al., 2023; Muñoz Martínez et al., 2024) included pre-service teachers. Some of the included studies referred to different disabilities (cognitive, sensory, and motor) and special educational needs of students; others focused on specific conditions such as specific learning disorders, ASD (autism spectrum disorder), EBDs (emotional and behavioral disorders), ADHD (attention deficit hyperactivity disorder), and disruptive behavior. Only one study (Gómez-Arizaga et al., 2016) referred to gifted students.

The studies included in this systematic review were based on different and partially overlapping definitions of the construct of emotional competence (EI, emotional awareness, emotional support, empathy, emotional regulation, emotional labor); moreover, they considered multiple indicators of inclusion and obtained specific results for the different disabilities and SENs studied. Below, the results are presented in relation to the aspects of emotional competence examined in the different studies, highlighting the inclusion indicators considered each time.

### 3.2. Emotional Intelligence and Inclusion

A first group of quantitative studies (Arias-Pastor et al., 2023; Dallasheh et al., 2021; Nwosu et al., 2023; Skura & Świderska, 2022) examined the global construct of EI, which was derived from the theory of Mayer and Salovey (1997) and defined as the ability to perceive and regulate one’s own emotions as well as the emotions of others and to use emotional information in social relationships.

Two studies (Arias-Pastor et al., 2023; Nwosu et al., 2023) investigated relationships between EI and teachers’ attitudes, concerns, and sentiments about inclusive education. According to Forlin et al. (Forlin et al., 2011), attitudes are teachers’ feelings towards the placement of children with SENs in mainstream classes, concerns refer to difficulties in coping with the demands of inclusive education, and sentiments refer to discomfort in social interactions with students with disabilities/SENs.

Arias-Pastor et al.’s (2023) study was conducted with pre-service teachers and showed that, in particular, the components of EI related to emotion regulation and empathy are associated with more favorable attitudes, concerns, and sentiments towards inclusion. In addition, EI is associated with greater attention to student diversity as well as better teacher well-being.

In contrast, Nwosu et al.’s (2023) study of secondary school teachers of students with diverse disabilities and SENs found that higher EI was associated with more positive attitudes but also greater concerns about inclusion, while there was no significant relationship with sentiments toward inclusion. The authors hypothesized that higher EI would lead teachers to be more aware of their difficulties and less confident in addressing them. Furthermore, they hypothesized that cultural factors play an important role, especially in a country like Nigeria, where the study was conducted and where there is still high resistance to inclusion.

The positive role of teachers’ EI for inclusion was also emphasized in the studies by Dallasheh et al. (2021) and Skura and Swiderska (2022). The first study focused on elementary and middle school teachers of students with learning disorders, while the second focused on elementary school teachers of students with various disabilities/SENs. In both studies, teachers with higher EI were found to report better classroom management and teacher–student relationships, higher student engagement and participation, and better academic and social inclusion.

### 3.3. Emotional Competence, Emotional Awareness, Emotional Support, and Inclusion

Three studies considered multidimensional constructs similar to EI, namely emotional competence (Silva & Marin, 2019), emotional awareness (Samnøy et al., 2023), and emotional support (Mudhar et al., 2024).

The construct of emotional competence encompasses emotional awareness, regulation of emotions, emotional expressiveness, and perception of emotions (Silva & Marin, 2019). This study involved teachers of students with mental and intellectual deficits and found that higher teacher emotional competence was associated with a better teacher–student relationship quality and better teacher ability to manage stress in the classroom, resulting in a better classroom climate.

The qualitative study by Samnøy et al. (2023) involved primary and secondary teachers of students with different special educational needs and defined emotional awareness, similar to EI, as the ability to recognize when emotions are present in oneself and others; to be attentive to emotional expressions, one’s own and those of others; to be attentive to what might provoke an emotional response; and to recognize the impact emotions can have on oneself and others. The study found that teachers’ emotional awareness was linked to a better school climate and to the well-being of teachers and students.

Finally, the construct of teacher emotional support (Mudhar et al., 2024) encompasses the connection between teacher and student, awareness of student needs, and consideration of the student perspective. The cited study involved secondary school teachers working with students with a range of SENs. Stronger emotional support from teachers was related to higher student engagement and a better classroom climate and teacher–student relationships. Furthermore, the study highlighted the role of emotional support in the implementation of collaborative teaching practices, which are a key aspect of promoting inclusion.

The other studies included in the review focused on specific aspects of emotional competence, in particular, empathy (De Klerk & de Klerk, 2018; Gómez-Arizaga et al., 2016; Graziano et al., 2024; Muñoz Martínez et al., 2024; Orozco & Moriña, 2023), emotion regulation (Hernández González et al., 2024a, 2024b; Koenen et al., 2019; McGrath & Van Bergen, 2019; Spilt et al., 2021), and emotional labor (Stark & Ragunathan, 2022; Valenti et al., 2019), and their findings will be illustrated in the following sections.

### 3.4. Empathy and Inclusion

DeKlerk and de Klerk (2018) in their qualitative study interviewed teachers of students with various disabilities and showed that empathy was related to the implementation of a supportive learning environment based on differentiated and creative teaching approaches, leading to higher student motivation.

The qualitative study by Gómez-Arizaga et al. (2016) was the only one that focused on gifted students and included both students’ and teachers’ perspectives, examined through focus group discussions. Teacher empathy is considered one of the factors related to student engagement, participation, and motivation, as well as an effective classroom climate. In addition, teacher empathy is linked to the ability to implement interdisciplinary teaching approaches.

The qualitative study by Orozco and Moriña (2023) of early childhood, primary, and secondary teachers of students with different SENs highlights that teacher empathy is among the factors related to the use of active teaching methods and inclusive planning, as well as continuous teacher training.

Similarly, in the qualitative study by Muñoz Martínez et al. (2024) with primary school teachers specializing in special education, empathy is highlighted as one of the key elements to promote inclusion. Empathy enables a deep understanding of students’ personal experiences and thus fosters an educational environment of mutual understanding and sensitivity to diversity. According to the participants, empathy is a key factor in building meaningful connections and trust in educational settings, thereby promoting learning outcomes and students’ emotional well-being.

Unlike all these studies, the study by Graziano et al. (2024) problematizes the positive role of empathy for inclusion: they found that higher levels of empathy were related to higher levels of self-efficacy in inclusive education, especially when levels of emotional self-efficacy were higher. In other words, high levels of empathy are not necessarily associated with greater ability to implement inclusive education, as they can lead to excessive emotional engagement and stress, which negatively affect teachers’ abilities. Therefore, empathy can foster the implementation of an inclusive approach to education, especially when teachers feel able to regulate negative emotions. Moreover, this relationship was only found for female teachers, which highlights that future research should also investigate the role of these factors in relation to the genders of teachers.

### 3.5. Emotion Regulation and Inclusion

A group of studies focused on the teacher’s ability to regulate emotions when teaching students with emotional and behavioral disorders (Koenen et al., 2019; Spilt et al., 2021), disruptive behavior (McGrath & Van Bergen, 2019), and autism spectrum disorder (ASD) (Hernández González et al., 2024a, 2024b). Students with these problems usually have difficulties with emotion regulation and may therefore exhibit challenging behaviors. Therefore, in their relationship with these students, teachers need to have skills in emotional self-regulation as well as in regulating the emotionality of the students themselves.

The quantitative study by Koenen et al. (2019) focused on teachers’ daily negative emotions when teaching students with emotional and behavioral disorders (EBDs) and attachment problems. Teachers’ ability to regulate negative emotions and manage their emotional exhaustion and risk of depersonalization was associated with better classroom management and a higher quality of teacher–student interactions. In addition, teachers’ ability to manage negative emotions was related to students’ behavioral adjustment and teachers’ emotional well-being.

The qualitative study by Spilt et al. (2021) was based on analyzing dialogs about emotions between elementary school teachers and their students with EBDs, while also examining students’ perspectives. The teacher’s ability to have coherent dialogs with these students, manage their hostility and negative emotions, and guide them through inclusion, structuring, and acceptance plays a crucial role in co-regulating the children’s emotions. Furthermore, the teacher’s emotion regulation has been shown to be related to the quality of teacher–child interactions as well as to children’s prosocial behavior.

The qualitative study by McGrath and Van Bergen (2019) involved classroom and special education teachers of students with disruptive behavior and found that teachers’ emotion regulation along with emotional awareness and empathy was not only related to improved student–teacher relationships and classroom climate but also to reduced student aggression and better behavior management.

Finally, in the quantitative study by Hernández González et al. (2024a, 2024b) involving elementary school teachers working with students with autism spectrum disorder (ASD), teachers’ emotion regulation strategies (especially cognitive reappraisal and expressive suppression of emotions) were found to be related not only to the quality of teacher–student interaction and the classroom climate but also to students’ behavioral adjustment. In addition, a greater ability to regulate emotions on the part of the teacher helps them to manage the relationship with students with ASD who exhibit anxiety-related problems. Thus, again, teachers’ emotion regulation has positive effects on inclusion linked to changes in both student behavior and classroom variables.

### 3.6. Emotional Labor and Inclusion

Both the studies by Stark and Ragunathan (2022) and Valenti et al. (2019) refer to a construct similar to emotion regulation called emotional labor, grounded in organizational psychology and defined as the work of reducing dissonance between one’s naturally experienced emotions and the emotional displays expected of an employee in their work environment (Hochschild, 2012). Emotional labor thus refers to how people manage and display their emotions while at work and how this influences performance, social dynamics at work, and well-being (Grandey et al., 2017). According to these authors (Stark & Ragunathan, 2022; Valenti et al., 2019), the construct of emotional labor is particularly meaningful for teachers, and especially for those who deal with students with EBDs, due to the difficulties in managing their own emotions and those of their students in the school context.

The quantitative study by Valenti et al. (2019) examined emotional labor by assessing two key constructs: emotional display rules (EDRs) and emotional acting (EA). Emotional display rules refer to the expectations for expressing or restraining emotions in a particular work context; for example, according to the EDRs, teachers should not raise their voices in school when they are upset with a student. Emotional acting refers to strategies used by teachers to meet the required emotional expressions in their professional context. For example, a teacher may be frustrated with a student, but the school’s EDR prohibits them from showing their frustration. To comply with this rule, the teacher may simply hide their feelings (i.e., surface acting) (where the teacher suppresses the expression of their frustration even though they are angry with a student). Alternatively, the teacher may change the emotion felt internally (i.e., deep acting) (where the teacher feels frustrated but recognizes the student’s efforts, transforms the frustration into appreciation, and expresses this to the student). Although deep acting may be considered a more adaptive strategy in classrooms, the study by Valenti et al. (2019) found that surface acting is a successful strategy used by special education teachers in self-contained classes to build a better working alliance with students with emotional and behavioral disorders and to promote their engagement in school tasks. According to the authors, teacher suppression of negative emotions may be the most appropriate strategy to maintain control during the most difficult moments of interaction with these students (e.g., escalating anger).

In the qualitative study by Stark and Ragunathan (2022), the construct of emotional labor was explored in depth using interviews with in-service educators working with students with EBDs. School is a professional context where there are often no explicit messages about what emotions to display professionally. Emotional labor is a continuous process through which teachers learn how to regulate the display of their emotions during interactions with students. To achieve this goal, it is important that teachers observe students and collect data on their emotional needs and competencies, reflect on their own emotions, share experiences with colleagues, and define their roles and responsibilities within the organizational system. Teachers’ ability to regulate emotions is crucial for promoting their students’ emotional self-regulation and academic achievement, as well as for improving the quality of the teacher–student relationship.

## 4. Discussion

The aim of this systematic review was to summarize the existing literature on the role of teachers’ emotional competence in promoting inclusive learning environments. Two complementary aspects were examined, namely the psychological constructs related to emotional competence that are relevant to inclusion according to the scientific literature and the aspects of inclusive education that are influenced by teachers’ emotional competence.

Eighteen studies were included in this systematic review, with the number of papers increasing from 2021 onwards. This result suggests that there is still relatively little research interest in the relationship between teachers’ emotional competence and inclusive education. However, it can be assumed that the growing interest is related to the requirements of the Convention on the Rights of Persons with Disabilities (CRPD) and the increasing awareness of the importance of this topic for education.

The geographical origin of the studies was quite spread, but half of the studies came from European countries. This result suggests that the topic of inclusion in relation to emotional competence is probably not yet fully understood worldwide, often in conjunction with highly differentiated national legislation on the subject. The different definitions of teachers’ roles found in the included studies (e.g., mainstream/general teachers, special education teachers, support teachers, educators/instructors) were also guided by the different normative frameworks of national school systems. This reflects the heterogeneous global landscape of inclusive education, in which different policies influence the responsibilities of teachers.

In most of the studies we included, the focus was on specific student diagnoses, which contradicts the CRPD principle of embracing diversity as a human right. Effective inclusion requires a move away from diagnosis-based approaches towards recognizing individual variability and addressing diversity in the classroom as a whole. Yet there is limited research on how the organizational context supports teachers’ inclusive efforts, suggesting a need to shift the focus from individual characteristics to systemic factors that enable inclusion.

Regarding the operational definition of teachers’ emotional competence in the included studies, we found different and partially overlapping psychological constructs; the most considered were EI, empathy and emotional support, emotional regulation, and emotional labor.

EI appears to be related to more positive attitudes and lower concerns about inclusion (Arias-Pastor et al., 2023; Dallasheh et al., 2021; Skura & Świderska, 2022), except in one study (Nwosu et al., 2023), where it was associated with greater concerns about the implementation of inclusive education. We believe that this finding highlights the crucial role of context: when the cultural and social characteristics of the context hinder inclusion (e.g., by labeling and marginalizing students with difficulties), teachers with higher EI and better inclusive attitudes also have more concerns about their work.

Studies looking at teachers’ empathy (De Klerk & de Klerk, 2018; Gómez-Arizaga et al., 2016; Graziano et al., 2024; Muñoz Martínez et al., 2024; Orozco & Moriña, 2023) and emotional support (Mudhar et al., 2024) emphasize the importance of both aspects in understanding and sharing students’ emotional experiences. Specifically, empathy enables teachers to improve relationships with students and the classroom climate, while emotional support appears to be important for the implementation of collaborative teachers’ practice. These two aspects of emotional competence therefore have an impact on the contextual characteristics of the classroom as well as a positive effect on teachers’ well-being. As claimed in the study by Graziano et al. (2024), empathy can promote the implementation of an inclusive approach to education, especially when teachers feel able to regulate negative emotions. Therefore, the crucial role of emotion regulation skills in relation to feelings of competence in inclusion is emphasized and a possible synergistic effect of empathy and emotional self-efficacy is outlined.

Finally, emotional regulation (Hernández González et al., 2024a, 2024b; Koenen et al., 2019; McGrath & Van Bergen, 2019; Spilt et al., 2021) and emotional labor (Stark & Ragunathan, 2022; Valenti et al., 2019) are most closely associated with teaching students with certain types of disabilities and with classroom management. Teachers who are more competent in emotion regulation are better able to deal with students with emotional and behavioral disorders, as this competence allows them to regulate both their own emotions and those of the student. In particular, emotional labor is a real working tool for the teacher who, by learning specific techniques for expressing emotions, helps students with difficulties to have a better school experience. Although the studies included show that emotional regulation and emotional labor are particularly useful in the management of students with disabilities, they are fundamental to the relationship with all students and the management of the class as a whole.

The present study has some limitations. First, some relevant studies may have been inadvertently omitted, although this risk was controlled by searching multiple databases and including a wide range of keywords related to emotional competence. Second, only papers published in peer-reviewed English journals were deliberately included in this review. For this reason, potentially relevant studies published in other languages may have been omitted, thereby losing information about possible cultural differences related to inclusive education. Third, the limited number of studies and the heterogeneity of psychometric measures of emotional competence prevented a meta-analysis from being conducted. Finally, this paper aimed to highlight the current state of knowledge about teachers’ emotional competence in relation to inclusive educational practices by analyzing studies from countries with very different cultures and educational systems. These differences may account for the concepts analyzed and the research findings of the studies included in the review. In any case, the key element that emerges from all the studies is the centrality of the emotional dimension in teaching and the ability of teachers to manage it.

## 5. Implications

Despite these limitations, this systematic review has implications for pre-service teacher education and in-service teacher training. Teaching is a relational profession with high emotional involvement, especially when the teacher has to support the development of students with disabilities or SENs. Teacher training should not ignore this aspect but should rather include specific training to develop appropriate emotional competence. This is even more necessary in the training of support teachers, who are in fact more exposed to critical conditions as they spend more time with students in difficulty; such teachers thus run a greater risk of psychological suffering and burnout (De Stasio et al., 2024).

However, pre-service and in-service training is not in itself a sufficient prerequisite for the emotional competence of teachers, and in particular of support teachers, to promote inclusive education. Teachers work in a social context that is rich in relationships, with students, colleagues, and all non-teaching staff in the school. The organizational context of the school plays a central role in the implementation of effective strategies for the management of teacher–student relationships. Unfortunately, we found no studies in the literature that addressed the role of the school organization in the development and maintenance of teachers’ emotional competence for inclusive education. Certainly, the characteristics and personal training of teachers constitute an indispensable condition for the effective performance of their profession, but only the organizational work context can ensure that educational strategies in general, and in particular those focused on the emotional dimension of the educational relationship, are systematic and effective (Engeström, 2008).

In the difficult process of school teaching and learning, which involves many teachers and even more students (all with different and sometimes very specific characteristics), we consider it crucial to shift the focus from the individual to the context; therefore, we would like to see new research that deepens the role of networking in schools with regard to the inclusion of all students, with and without disabilities or SENs.

## 6. Conclusions

The increasing attention paid to aspects of the emotional dimension of the teaching profession underlines the growing awareness of the centrality of this dimension to that profession, particularly when it comes to supporting teachers. The published studies that have addressed this topic refer to different concepts, but these can usually be traced back to the more general construct of emotional competence. The most studied aspects concern the ability to correctly recognize one’s own emotions and those of others, to know how to regulate and control one’s own emotional state, and to make it useful in existing social relationships (especially with students with special difficulties). These skills have proven to be essential for successful teaching, both from a strictly didactic point of view (they improve students’ performance in school subjects) and in terms of the development of students’ emotional and social skills, especially those who face systemic barriers to learning. Moreover, these findings have significant implications for educational policymaking. Given the centrality of teachers’ emotional competence in fostering inclusive education, policymakers should consider integrating structured emotional competence training within teacher education programs and professional development initiatives. Additionally, school-wide policies should promote institutional support mechanisms that help educators navigate the emotional challenges of inclusive teaching, ultimately contributing to more effective and sustainable inclusion strategies (Dolev & Leshem, 2017; Lozano-Peña et al., 2021; OECD, 2024).

## Figures and Tables

**Table 1:** Studies included.

Author and Year	Country	Research Design	Sample/Grade Level	Type of Disability/SEN	Critical Aspects of Teacher Emotional Competence	Impacted Elements of Inclusive Education	Synthesis of Study Results
Arias-Pastor et al., 2023	Spain	Empirical descriptive, quantitative, and cross-sectional research approach, utilizing a questionnaire as the primary instrument for data collection	218 pre-service teachers (MDSE students) from various Spanish universities (Mage = 31.5; SD = 6; males = 33%; females = 67%)	Various special educational needs (SENs)	EI (emotional awareness, emotional regulation, empathy), motivation, collaborative skills	Teacher attitudes and concerns about inclusion and sentiments towards students with disabilities, facilitation of inclusive education processes, quality attention to diversity in classrooms and improved teacher well-being	EI has a positive effect on both teacher well-being and the facilitation of inclusive education processes and diversity attention
Dallasheh et al., 2021	Israel	Quantitative correlative design, utilizing self-report questionnaires evaluating EI, learning motivation, and school inclusion ability	406 special education in-service teachers (128 male and 278 female; 51% under 40 years old, 49% over 40 years old) working in elementary and middle schools within the Arab minority in Israel	Specific learning disorders	EI (emotional awareness and regulation, empathy), ability to build positive relationships, effective communication	Classroom management and environment, student engagement and participation, academic and social inclusion, individualized support and accommodations	Teachers’ EI was positively related to students’ learning motivation. This relationship was moderated by teachers’ inclusion capability. Higher EI was reported by females, younger teachers, and teachers with fewer years of service
De Klerk & de Klerk, 2018	South Africa	Qualitative, phenomenological research design, utilizing in-depth interviews to explore educators’ experiences of empathy within inclusive classrooms	7 female in-service educators (Mage = 48.4) from three inclusive schools in the Dr. Kenneth Kaunda District, North West Province	Various disabilities including visual, hearing, cognitive, and physical disabilities	Intrapersonal proficiency, interpersonal understanding, adaptive teaching skills, situational empathy	Creation of a supportive learning environment, development of trust, motivation and acknowledgment of learners, differentiation and creative teaching approaches	Teachers’ empathy allows them to understand and motivate learners with disabilities and to adapt their teaching skills in relation to the different needs of the learners
Gómez-Arizaga et al., 2016	Chile	Qualitative design using focus groups to analyze perceptions of community members about critical competencies of instructors working with gifted students	6 focus groups with 18 participants (9 males, 9 females): students, in-service instructors, and staff members of an enrichment program for gifted students	Gifted students	Knowledge (disciplinary preparation, passion for content), teaching (flexibility, student participation, motivation), socio-emotional characteristics (empathy, closeness, passion, reflection)	Effective classroom climate, student engagement and participation, development of trust and motivation, interdisciplinary teaching approaches	Empathetic teachers are capable of understanding the emotions expressed by their students, they know how to treat them, and they demonstrate a profound sense of respect toward the student as an individual
Graziano et al., 2024	Italy	Quantitative, cross-sectional	739 in-service support teachers (86.9% female; Mage = 37.7, SD = 8.4) from primary and secondary schools	Various disabilities and SENs	Empathy and emotional self-efficacy	Self-efficacy for inclusive education	Higher levels of empathy were related to higher levels of self-efficacy in inclusive education, especially when levels of emotional self-efficacy were higher. This relationship was only found for female teachers
Hernández González et al., 2024a	Chile	Descriptive, comparative, correlational, cross-sectional	139 in-service teachers from inclusive primary and preschools (83% female, 84% >30 years old)	Autism spectrum disorder (ASD) and anxiety disorders	Emotion regulation (cognitive reappraisal and expressive suppression of emotions), ASD awareness	Quality of teacher–student interactions, teachers’ emotional well-being, classroom climate, student behavioral adjustment	Cognitive reappraisal of emotions is related to teachers’ responses that promote ASD students’ autonomy; expressive suppression of emotions is related to ASD students’ anxiety responses
Hernández González et al., 2024b	Chile	Descriptive, comparative, correlational, non-experimental, cross-sectional	131 in-service teachers from primary and preschools (82% female; 12% under 30, 70% between 31 and 50; 18% over 50 years old)	Autism spectrum disorder (ASD)	Emotion regulation (cognitive reappraisal and expressive suppression of emotions)	Participation of students with ASD in school activities, teacher–student relationship, classroom management	Teachers’ cognitive reappraisal of emotions was related to higher participation of ASD students in school
Koenen et al., 2019	Belgium	Quantitative, collecting daily data (diary method) on teachers’ negative emotions in interactions with individual students with attachment problems over a 3-week period	58 in-service teachers (12% male; Mage = 33.46; SD = 7.13) and 71 teacher–student dyads in special education primary schools for students with emotional and behavioral disorders	Emotional and behavioral disorders (EBDs), specifically students with attachment problems	Self-efficacy in classroom management, supportive teaching style, managing emotional exhaustion, avoiding depersonalization	Quality of teacher–student interactions, teachers’ emotional well-being, classroom climate, student behavioral adjustment	Teachers with high self-efficacy in classroom management and a highly supportive teaching style were less likely to experience negative emotions (angry, irritated, tense, guilty, helpless, sad)
McGrath & Van Bergen, 2019	Australia	Qualitative study, using a teacher speech sample task to examine relational closeness between elementary teachers and disruptive students	11 classroom in-service teachers (2 males, years of experience M = 12.3, SD = 10.7), 7 support in-service teachers (all females, years of experience M = 15.6, SD = 11.8), and 8 elementary students from three Australian government schools in Sydney	Disruptive behavior among students	Emotional awareness, empathy, emotion regulation, emotional perspective-taking	Student–teacher relationships, classroom climate, student aggression, behavior management	Teachers who were more aware of students’ emotions, who expressed empathy, and who used this information for effective classroom management were more likely to express greater relational closeness to disruptive students
Mudhar et al., 2024	Norway	Mixed-methods cluster randomized controlled trial, utilizing self-report online surveys to investigate patterns of teachers’ self-efficacy and attitudes toward inclusive education	100 upper secondary school in-service teachers (62% female, Mage = 43.10, SD = 8.87) from 12 schools across two Norwegian counties	Various special educational needs (SENs)	Teacher emotional support (teacher–student connection, awareness of students’ needs, regard for student perspectives), collective teacher efficacy, collegial collaboration	Classroom climate, student engagement, teacher–student relationships, collaborative teaching practices	Teachers reporting high teacher self-efficacy and high positive attitudes toward inclusive education were the most emotionally supportive toward students
Muñoz Martínez et al., 2024	Spain	Qualitative approach grounded in Participatory Action Learning and Action Research	14 primary education teachers (11 F, 3 M; Mage = 23.6) specializing in special educational needs	Various special educational needs (SENs)	Respect for differences, empathy, motivation, positive attitude, teacher collaboration, self-reflection coupled with problem-solving skills	Students’ learning and participation, mutual understanding, students’ well-being	Teachers in this study considered empathy one of the key attributes for inclusive and socially just teaching
Nwosu et al., 2023	Nigeria/ South Africa	Correlational research design, utilizing hierarchical regression analysis to investigate associations between EI and teachers’ attitudes, concerns, and sentiments about inclusive education	508 general classroom in-service teachers (78% female, Mage = 36.7, SD = 9.8) in public secondary schools in Onitsha, Anambra State	Various disabilities including learning disabilities, ADHD, and ASD (autism spectrum disorders)	EI (emotional awareness, empathy, self-regulation, social skills)	Teacher attitudes towards inclusion, concerns about handling inclusive education, teacher sentiments towards students with disabilities	Teacher EI was positively associated with attitudes and concerns about inclusive education, whereas no relationship emerged with sentiments about inclusive education
Orozco & Moriña, 2023	Spain	Qualitative multi-case study, using semi-structured interviews to explore teachers’ recommendations for inclusive education practices	100 Spanish in-service teachers (77% female, Mage = 45.3) from early childhood, primary, secondary, and higher education	Various special educational needs (SENs)	Emotional awareness, empathy, motivation, reflection, collaborative skills	Inclusive planning, active teaching methodologies, ethical and emotional competencies, ongoing teacher training	Teachers’ empathy, communication skills and ability to build respectful relationships with students are related to inclusion
Samnøy et al., 2023	Norway	Qualitative study, using focus groups to explore teachers’ experiences of a Continuing Professional Development (CPD) program focused on enhancing emotional awareness	22 primary and secondary school in-service teachers (16 F, 6 M; years of teaching experience M = 21) in four schools in Norway	Various special educational needs (SENs)	Emotional awareness, empathy, reflection, sensitivity to emotional dynamics	Teacher well-being, student well-being, classroom climate, professional competence in handling emotional dimensions	The teachers in this study considered learning about emotional awareness relevant and useful for their everyday lives as teachers, especially in terms of making them more aware of the significance of emotions in the school context
Silva & Marin, 2019	Brazil	Observational–analytical, quantitative and cross-sectional study using surveys to assess emotional competence and coping styles in teachers	63 in-service teachers (98.4% female, Mage = 43.1, SD = 8.30) from schools in São Leopoldo-RS, teaching students with deficits in mental/intellectual functions	Mental/intellectual deficits	Emotional competence (emotional awareness, regulation of emotions, emotional expressiveness, perception of emotions), adaptive coping strategies	Quality of teacher–student relationship, teacher’s ability to handle classroom stress, overall classroom climate	Emotional competence and some coping styles, such as not criticizing oneself for what happens in stressful situations, are fundamental for the quality of the student–teacher relationship. The inability to deal with emotions was related to frustration, discouragement, and increasing conflict
Skura and Świderska, 2022	Poland	Quantitative study, using the Two-dimension EI Inventory and the Social Competences Questionnaire to measure EI and social competences in teachers working with SEN students	225 primary in-service schoolteachers in Warsaw, including 64 from mainstream schools, 97 from integrative schools, and 64 from special schools (F = 194, M = 30; <40 years = 116; >40 years = 108)	Various disabilities including intellectual disabilities, ASD (autism spectrum disorder), physical disabilities, hearing impairments, visual impairments, and mental illnesses	EI (emotional awareness, empathy), social competences, assertiveness	Teacher’s effectiveness in handling SEN students, teacher–student relationships, classroom management, social integration	Teachers with higher EI experienced fewer difficulties in working with the particular groups of students with SEN. The authors conclude that the specifics of working with students with SEN require teachers to have highly developed EI
Spilt et al., 2021	Belgium	Descriptive study, utilizing the Autobiographical Emotional Events Dialogue (AEED) coding system to assess teacher–child emotion dialogues	85 children and 70 in-service teachers (90% female; Mage = 34.5, SD = 8.08) from special education schools serving children with emotional and behavioral disturbances	Emotional and behavioral disturbances (EBDs)	Emotional awareness, empathy, teacher guidance, resolution of negative emotions, co-regulation skills	Quality of teacher–child interactions, teacher–child relationship quality, children’s prosocial behavior, emotional self-regulation	The quality of teachers’ dialogue about negative emotional events was related to children’s prosocial behavior and to better teacher–child relationship quality
Stark & Ragunathan, 2022	USA	Case study, utilizing thematic analysis of open-ended interviews to explore educators’ emotional experiences in an alternative program for students with emotional and behavioral disabilities (EBDs)	11 in-service educators working in an alternative high school program for students with EBDs (F = 7, M = 4; age n/a)	Emotional and behavioral disabilities (EBDs)	Emotional regulation, strategic emotional expression, emotional labor, empathy, gathering data on students’ emotional needs	Student emotional development, teacher–student relationship quality, student self-regulation, academic and socio-emotional outcomes	Educators’ strategic use of their emotional expressions to serve students is related to better teacher–student relationships and better student self-regulation
Valenti et al., 2019	USA	Empirical study, using multilevel path analyses to estimate mediational effects of emotional display rules and emotional acting on teacher–student working alliance	61 special education in-service teachers (75% female; Mage = 32) serving K-12 students with EBDs in self-contained classrooms in Western Pennsylvania	Emotional and behavioral disorders (EBDs)	Emotional labor, surface acting, deep acting, perception of emotional display rules	Teacher–student working alliance, emotional regulation, quality of educational tasks, student engagement, behavioral outcomes	Research has shown that educators who are more socially and emotionally competent are more likely to create nurturing relationships and high-quality classroom environments, which result in more academic success for students

## Data Availability

No new data were created or analyzed in this study.
